# High degree of efficacy in the treatment of cyclic vomiting syndrome with combined co-enzyme Q10, L-carnitine and amitriptyline, a case series

**DOI:** 10.1186/1471-2377-11-102

**Published:** 2011-08-16

**Authors:** Richard G Boles

**Affiliations:** 1 Division of Medical Genetics and the Saban Research Institute, Childrens Hospital Los Angeles, California, USA; 2 Department of Pediatrics, Keck School of Medicine at the University of Southern California, Los Angeles, California, USA

## Abstract

**Background:**

Cyclic vomiting syndrome (CVS), defined by recurrent stereotypical episodes of nausea and vomiting, is a relatively-common disabling and historically difficult-to-treat condition associated with migraine headache and mitochondrial dysfunction. Limited data suggests that the anti-migraine therapies amitriptyline and cyproheptadine, and the mitochondrial-targeted cofactors co-enzyme Q10 and L-carnitine, have efficacy in episode prophylaxis.

**Methods:**

A retrospective chart review of 42 patients seen by one clinician that met established CVS diagnostic criteria revealed 30 cases with available outcome data. Participants were treated on a loose protocol consisting of fasting avoidance, co-enzyme Q10 and L-carnitine, with the addition of amitriptyline (or cyproheptadine in those < 5 years) in refractory cases. Blood level monitoring of the therapeutic agents featured prominently in management.

**Results:**

Vomiting episodes resolved in 23 cases, and improved by > 75% and > 50% in three and one additional case respectively. Among the three treatment failures, two could not tolerate amitriptyline (as was also the case in the child with only > 50% efficacy) and one had multiple congenital gastrointestinal anomalies. Excluding the latter case, substantial efficacy (> 75% response) was 26/29 at the start of treatment, and 26/26 in those able to tolerate the regiment, including high dosages of amitriptyline.

**Conclusion:**

Our data suggest that a protocol consisting of mitochondrial-targeted cofactors (co-enzyme Q10 and L-carnitine) plus amitriptyline (or possibly cyproheptadine in preschoolers) coupled with blood level monitoring is highly effective in the prevention of vomiting episodes.

## Background

Cyclic vomiting syndrome (CVS) is characterized by recurrent identical episodes of nausea and vomiting, with the absence of these symptoms between episodes [1]. CVS is likely common, being present in about 2% of Scottish [2] and Western Australian [3] school children. Prior to the advent of successful therapy, CVS was a disabling condition as episodes are generally severe, usually last for days, and often require intravenous fluid therapy for dehydration [1]. Frequent and prolonged school or work absences lead to academic or work disability. CVS can pose a challenge for clinicians to manage, and it is common for patients to seek help from multiple practitioners because of continued vomiting episodes.

Although the etiology is unknown, substantial parallels with migraine headache [4] have prompted therapeutic trials with anti-migraine therapies. Amitriptyline (Elavil^®^), a tricyclic "antidepressant" frequently used to treat migraine, is the most widely prescribed prophylactic medication used for the treatment of CVS, with response rates varying from 52-73% in open-label and subject recall-based studies in children and adults [reviewed in 5]. In a recent consensus statement, amitriptyline was recommended as the first-line treatment choice for CVS prophylaxis in children and adolescents age 5 years and older, while cyproheptadine is recommended in younger children [1].

Mitochondrial dysfunction is hypothesized to be a factor in the pathogenesis of both CVS and migraine headache based upon decreased respiratory complex enzymology, disease-associated mitochondrial DNA (mtDNA) sequence variants, and preferential maternal inheritance [reviewed in 5]. Physicians and other health care providers are increasingly recommending co-enzyme Q10, also known as ubiquinone, a commonly-used dietary supplement that is widely available in retail settings, for the treatment of a wide variety of conditions, including mitochondrial dysfunction. Co-enzyme Q10 serves as the electron shuttle between complexes 1 or 2 and complex 3 of the mitochondrial respiratory chain [6]. In migraine, a randomized control trial demonstrated therapeutic efficacy [7]. Recently, the use of co-enzyme Q10 has been gaining in popularity among CVS patient groups. A recent subject recall-based study in CVS suggested equivalent efficacy of co-enzyme Q10 and amitriptyline (~70%), but with superior tolerability of co-enzyme Q10 [5]. There was inadequate data to assess response to combination therapy with both agents.

L-carnitine is also a naturally-occurring dietary supplement that is frequently used in the treatment of mitochondrial dysfunction [6]. L-carnitine is a shuttle of long-chain fatty acids across the inner mitochondrial membrane and thus is required for fat oxidation. In addition, L-carnitine has a "detoxifying" role in shuttling accumulated intermediates of metabolism out of impaired mitochondria. One case series [8] demonstrated efficacy of L-carnitine in CVS prophylaxis.

In the author's clinical experience, episodes of nausea and vomiting diminish markedly in the vast majority of CVS patients treated with a protocol consisting of fasting avoidance, co-enzyme Q10, and L-carnitine, with the addition of amitriptyline or cyproheptidine in refractory participants over and under the age of five years, respectively. One essential aspect of this protocol is dosing based on blood levels. The current study is a retrospective chart review of the 42 CVS patients evaluated over a two-year period by the author to evaluate therapeutic responses.

## Methods

A computer-generated report of all clinic patients seen by the author during the two-year period from 7/1/06 to 6/30/08 was reviewed for ICD 9 codes used by the author for CVS patients, including 536.2 and 277.87. A medical record review was performed on all cases so identified. Patients were included as participants in this study if given a diagnosis of CVS by the author based on fulfilling both the NASPGHAN [1] and Rome III [9] criteria. All participants are unrelated. All records were reviewed up until 6/30/10, allowing for a two-year follow-up period to access medium-term treatment responses. This study was approved by the Children's Hospital Los Angeles Institutional Review Board.

Participants were treated on a clinical basis, and not as part of a prospective study; however, treatment during this period was standardized as based on prior clinical experience and the literature [1]:

• Dietary: All subjects were advised to make dietary changes [1], including the "3+3 diet" (3 meals and 3 snacks a day including between meals and at bedtime), and the avoidance of fasting.

• Co-enzyme Q10: Participants were treated with co-enzyme Q10 (ubiquinone) in liquid or gel capsule form (from a variety of brands) at a starting dose of 10 mg/kg/day, or 200 mg, divided twice a day, whichever is smaller.

• L-carnitine: Participants were treated with Carnitor brand or generics at a starting dose of 100 mg/kg/day divided BID, or 2 grams twice a day, whichever is smaller. A small minority of families, all with untreated free carnitine blood levels > 30 micromolar, were not treated.

• Amitriptyline: Participants age 5 years and over with continued vomiting episodes despite the above therapies were treated at a starting dose of 0.5 mg/kg/day given at night An EKG was performed looking at the QTc interval prior to and a few weeks following starting treatment.

• Cyproheptadine: Participants under the age of 5 years with continued vomiting episodes despite the above therapies were treated at a starting dose of 0.25 mg/kg/day divided twice a day.

• Topiramate: Two participants who were refractory to all of the above measures were started on 25 mg of topiramate twice a day.

Dosages were increased every one to a few months until one of the following occurred:

• Resolution of vomiting episodes

• Intolerable side effects that failed a reduction in dosage followed by a slow dosage increase

• The following maximum was reached (empirically-derived):

ο Co-enzyme Q10: blood level > 3.0 mg/L

ο L-carnitine: free carnitine blood level > 40 micromolar

ο Amitriptyline*: amitriptyline + nortriptyline blood level > 150 ng/ml

ο Cyproheptadine: Dosage of 0.5 mg/kg/day

ο Topiramate Dosage of 200 mg twice a day (in adolescents and adults)

*Blood levels were not routinely monitored for dosages < 1 mg/kg/day as they were uniformly low in the authors' prior experience.

Efficacy was queried in terms of two parameters: episode frequency and episode duration. The efficacy category was determined by the percent improvement in the parameter demonstrating the greatest response at the time of the most-recent clinic visit prior to 6-30-10:

• Resolution (episodes resolved, allowing for one episode a year with an obvious trigger, usually a febrile infection).

• > 75% improvement (between 75-100% response in at least one episode parameter).

• > 50-75% improvement (between 50-75% response in at least one episode parameter)

• Treatment failure (< 50% improvement in both parameters)

## Results

A total of 42 participants met the study criteria. Age at the time of chart review varied from 3 to 26 years, with a median of 12 years. The age of the onset of vomiting episodes was 1 week to 15 years, with a median of 4 years. The female:male ratio was 2.2:1 (29 females and 13 males). The race/ethnicity was 28 (67%) Caucasians, 11 (26%) Hispanics, 2 (5%) African-Americans, and 1 (2%) Native-American. Several co-morbid, predominantly-"functional", conditions were common, ranging from zero (in two adults) to 16 per participant, with a median of 5.5 co-morbid conditions (Table 1).

**Table 1:** Chronic co-morbidities at 10% or greater prevalence among the 42 participants

Category or Condition	Number	Percent
**Chronic pain syndromes (any)**	**31**	**74%**

Extremity pain	17	40%

Headache (all but one with migraine)	16	38%

Abdomen	15	36%

Complex regional pain syndrome	5	12%

**Gastrointestinal dysmotility (any)**	**31**	**74%**

Gastroesophageal reflux disease and/or chronic nausea	23	55%

Colonic (irritable bowel syndrome, constipation, diarrhea)	17	40%

**Other functional or autonomic-related conditions (any)**	**24**	**57%**

Abnormal heart rate	11	26%

Abnormal temperature regulation	7	17%

Dizziness	6	14%

Tinnitus	5	12%

**Mental health disorders (any)**	**13**	**31%**

Depression	10	24%

**All cognitive disorders (any, including attention deficit)**	**15**	**36%**

Mental retardation or learning disabilities	13	31%

Autistic spectrum disorder	4	10%

**Other conditions**		

Any neuromuscular disorder (non cognitive)	18	43%

Chronic fatigue or exercise intolerance	23	55%

The inheritance pattern as estimated by Quantitative Pedigree Analysis [10] in the 35 cases with available data was 21 (60%) participants with probable maternal inheritance, 4 (11%) indeterminate, and 10 (29%) with probable non-maternal inheritance.

Nine participants were excluded from outcome analyses because they were seen in clinic only once or twice, and no follow-up data was available to determine their response to therapy, including five of the 10 adults (age > 18 years), but only 4 of the 32 children (*P *= 0.02). Two additional children were excluded because CVS resolved prior to starting therapy. One additional case was excluded because the parents declined prophylactic therapy and chose to continue to abort episodes with lorazepam and diphenhydramine.

Records in the remaining 30 subjects were queried for data related to treatment response (Table 2). This included three participants over the age of 18 years who were included in the study as they are of ages commonly treated by pediatricians, and the physiology of youth in their early to mid 20s is similar to that of adolescents.

**Table 2:** Response to treatment in 30 cases with cyclic vomiting syndrome

Amitriptyline	Cyproheptadine	Coenzyme Q10	L-carnitine	Outcome: Episodes Resolved	Outcome: Episodes > 75% Improvement	Outcome: Episodes > 50% Improvement	Outcome: Treatment Failure
		+	+	3	3^a^		

+				1			1 ^b^

+		+		3 ^c^			

+			+	2 ^d^			

+		+	+	10		1 ^b^	2 ^b^

	+			1			

	+	+		1			

	+		+	1			

	+	+	+	1			

^a ^All 3 families elected not to treat with amitriptyline despite symptoms

^b^See text

^c^In one of those cases, amitriptyline was not tolerated, yet episodes resolved on topiramate + co-enzyme Q10

^d^In one of those cases, episodes returned years later, then resolved on topiramate, while still on carnitine.

The treatment protocol failed in three cases, and was sub-optimal (50-75% response) in another case. In one of the treatment failure cases, episodes completely resolved for several months on amitriptyline alone. Unfortunately, a prolonged QTc interval was noted, which resolved on discontinuation without adverse events. Episodes then returned, but further therapy and evaluation were complicated by severe non-compliance. Two participants on amitriptyline, co-enzyme Q10, and carnitine had tolerance issues with amitriptyline. One of them (also labeled as treatment failure) demonstrated good efficacy, yet amitriptyline was discontinued because of narcolepsy, and episodes returned. In the other case (labeled as sub-optimal), behavioral and emotional effects have limited treatment to a sub-therapeutic amitriptyline level associated with only partial efficacy. In the final case of treatment failure, no improvement was noted on the same three treatments, as well as with the further addition of cyproheptadine. This latter infant has multiple malformations, including esophageal atresia, tracheoesophageal fistula, imperforate anus, and a tethered spinal cord, as part of VATER association, and thus was excluded from further data analyses.

Six participants reported side effects with amitriptyline. In addition to the three cases discussed above in which side effects necessitated treatment discontinuation or reduction, in three other participants side effects (increased frustration in two, one also with insomnia, dizziness in the other) did not limit treatment. One participant discontinued co-enzyme Q10 because of a pseudoporphyria rash. Such an association has not been reported, and the rash did not reappear on treatment with another brand of co-enzyme Q10. Cyproheptadine caused lethargy in one participant, and two had vague non-specific sensations while on multiple medications both related and unrelated to this study.

Urine ketosis was noted in the medical record as positive in 20 out of 20 cases tested during vomiting episodes. Ketosis was not seen at baseline.

## Discussion

This case series demonstrates excellent efficacy of cofactor therapy (co-enzyme Q10, L-carnitine) combined with amitriptyline. Treatment responses were suboptimal in only four cases, three of which could not tolerate adequate dosages of amitriptyline, and never achieved a "therapeutic" blood level (> 80 ng/ml of amitriptyline + nortriptyline). With the removal of the fourth case of the infant with multiple gastrointestinal malformations, substantial efficacy (> 75% response) of this protocol in children and youth > age 5 years was 19/22 at the onset of treatment, and 19/19 in participants able to tolerate amitriptyline. In the author's observations, making treatment decisions contingent of the blood levels of co-enzyme Q10, carnitine and amitriptyline was very helpful in many cases, as children with sub-optimal clinical improvement always demonstrated a low level of at least one of the three agents, and increased dosing was associated with the resolution of episodes. In order to achieve these "therapeutic" blood levels and clinical efficacy, some subjects required higher-than-customary dosages, including up to 25 mg/kg/day (800 mg a day in larger subjects) of co-enzyme Q10 and 2 mg/kg/day of amitripyline. These dosages were well tolerated.

In participants under age five years, efficacy appears to be good when cofactor therapy is combined with cyproheptadine, although the number of cases reported here is small. Drug treatment varied by age in the present study and in the NASPGHAN recommendations due to expert opinion regarding low tolerability (tachycardia and increased frustration) of amitriptyline in younger children and low efficacy of cyproheptadine in older children [1].

Combining the 22 cases > age 5 years and 4 cases < 5 years, overall substantial efficacy (> 75% response) of this protocol was 23/26 at the start of treatment, and 23/23 of those who could tolerate the regiment.

Clinical [11] and molecular [12] data suggest that CVS in adults, in particular with the adult onset of vomiting episodes [12] is distinct in many ways than CVS in children. However, among the five adult cases with outcome data in the present study, all of which had the adolescent onset of vomiting episodes, two did not tolerate amitriptyline (see footnote 5 in Table 2) and in the three others episodes resolved (two with all three agents, one with amitriptyline alone). Thus, there is inadequate data in this generally-young cohort to suggest alternative management based on adult age, although there may be a higher rate of intolerance to amitriptyline in adults than in children over age 5.

The major limitation on this study is that the participants were treated on the basis of best available clinical therapy, not on a prospective clinical trial. The protocol was used as guidelines, not on a rigorous basis. For example, participants with severe disease (multiple hospitalizations) were often treated simultaneously with cofactors and medication (amitriptyline or cyproheptadine) at the first visit based on the authors' experience of frequent treatment failures on cofactors alone, while those with milder disease courses were always given a trial of cofactors alone. Some families started the therapies sequentially, and once episodes stopped or greatly diminished would elect not to treat with agents not yet attempted. A few families declined co-enzyme Q10 therapy due to costs, which unlike all the other therapies in this report was rarely covered by insurance. A small number of participants were referred to the author with partial efficacy on amitriptyline or cyproheptadine, and when episodes resolved after increasing the dosage the families chose not to start one or both cofactors. These factors contributed to the complexity of the medical regiments as listed in Table 2. However this limitation does not diminish the observations herein of very-high efficacy in general using these agents in clinical practice, either alone or in combination.

The participants in this study include cases diagnosed by the author in a primary care-like setting, tertiary care cases referred by local pediatricians and gastroenterologists, and quaternary care cases from other states that failed multiple previous attempts at therapy. Since most participants were ascertained in the latter two situations, the present cohort is a sicker, more-treatment-resistant population of CVS than is likely to be encountered by all but a few practitioners. Since the more mildly-affected participants often responded well to cofactor therapy alone, and that the side effects of the cofactors are generally much less than that of the medications [5 and author's experience], a trial of cofactor and dietary therapy alone may be warranted in most CVS patients encountered in clinical practice, with amitripyline or cyproheptidine added in refractory cases.

Many participants discontinued therapy at some point, and in most the episodes returned, later resolving again on renewed therapy. In the exceptional cases, vomiting episodes evolved into migraine headache, often at the time of puberty, and the same protocol was used successfully in migraine prophylaxis. No participants are known to be off therapy and without both vomiting episodes and migraine in the medium-term follow-up period of this study.

## Conclusions

CVS is a disabling, common and difficult-to-treat condition. Our data suggest that a protocol consisting of mitochondrial-targeted cofactors (co-enzyme Q10 and L-carnitine) plus amitriptyline (or possibly cyproheptadine in preschoolers) coupled with fasting avoidance and blood level monitoring is highly effective in the prevention of vomiting episodes. A prospective blinded clinical trial is needed. However, given the suggestion of efficacy and excellent tolerability, health care providers may want to consider combining these cofactors as a low-risk therapeutic option along with the NASPGHAN recommendations of amitriptyline (> 5 years) or cyproheptadine (< 5 years). A trial first of cofactors and fasting avoidance alone may be warranted in cases without a history of multiple hospitalizations for vomiting episodes.

## Competing interests

The author declares that they have no competing interests.

## Authors' contributions

Not applicable, the sole author, RGB, performed all tasks.

## Pre-publication history

The pre-publication history for this paper can be accessed here:

http://www.biomedcentral.com/1471-2377/11/102/prepub
